# Butane-1,4-diaminium bis­[3,4,5,6-tetra­chloro-2-(meth­oxy­carbon­yl)benzoate]

**DOI:** 10.1107/S1600536811016795

**Published:** 2011-05-07

**Authors:** Zu Pei Liang

**Affiliations:** a Department of Chemistry and Chemical Engineering, Weifang University, Weifang 261061, People’s Republic of China

## Abstract

In the title salt, C_4_H_14_N_2_^+^·2C_9_H_3_Cl_4_O_4_^−^, the cation lies on an inversion center. In the anion, the mean planes of meth­oxy­carbonyl and carboxyl­ate groups form dihedral angles of 64.9 (3) and 58.5 (3)°, respectively, with the benzene ring. In the crystal, inter­molecular N—H⋯O hydrogen bonds connect the components into sheets parallel to (100).

## Related literature

For a related structure, see: Li (2011 ▶).
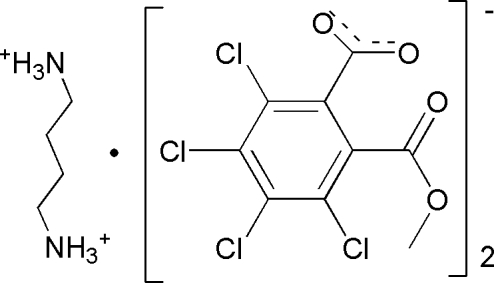


## Experimental

### 

#### Crystal data

C_4_H_14_N_2_^2+^·2C_9_H_3_Cl_4_O_4_^−^
*M* *_r_* = 724.00
Monoclinic, 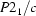

*a* = 14.4243 (13) Å
*b* = 6.1041 (6) Å
*c* = 16.9653 (15) Å
β = 97.056 (1)°
*V* = 1482.4 (2) Å^3^
*Z* = 2
Mo *K*α radiation
μ = 0.81 mm^−1^
*T* = 298 K
0.46 × 0.43 × 0.40 mm

#### Data collection

Bruker SMART CCD diffractometer
Absorption correction: multi-scan (*SADABS*; Bruker, 1997 ▶) *T*_min_ = 0.708, *T*_max_ = 0.738
7307 measured reflections
2603 independent reflections
1908 reflections with *I* > 2σ(*I*)
*R*_int_ = 0.024

#### Refinement

*R*[*F* ^2^ > 2σ(*F* ^2^)] = 0.036
*wR*(*F* ^2^) = 0.096
*S* = 1.05
2603 reflections
183 parameters
H-atom parameters constrained
Δρ_max_ = 0.23 e Å^−3^
Δρ_min_ = −0.20 e Å^−3^

### 

Data collection: *SMART* (Bruker, 1997 ▶); cell refinement: *SAINT* (Bruker, 1997 ▶); data reduction: *SAINT*; program(s) used to solve structure: *SHELXS97* (Sheldrick, 2008 ▶); program(s) used to refine structure: *SHELXL97* (Sheldrick, 2008 ▶); molecular graphics: *SHELXTL* (Sheldrick, 2008 ▶) and *PLATON* (Spek, 2009 ▶); software used to prepare material for publication: *SHELXTL*.

## Supplementary Material

Crystal structure: contains datablocks global, I. DOI: 10.1107/S1600536811016795/lh5244sup1.cif

Structure factors: contains datablocks I. DOI: 10.1107/S1600536811016795/lh5244Isup2.hkl

Supplementary material file. DOI: 10.1107/S1600536811016795/lh5244Isup3.cml

Additional supplementary materials:  crystallographic information; 3D view; checkCIF report

## Figures and Tables

**Table 1:** Hydrogen-bond geometry (Å, °)

*D*—H⋯*A*	*D*—H	H⋯*A*	*D*⋯*A*	*D*—H⋯*A*
N1—H1*C*⋯O3^i^	0.89	1.85	2.735 (3)	172
N1—H1*B*⋯O4^ii^	0.89	1.94	2.823 (3)	174
N1—H1*A*⋯O4	0.89	1.88	2.761 (3)	169

Symmetry codes: (i) 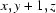; (ii) 
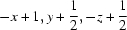
.

**Fig. 1.**
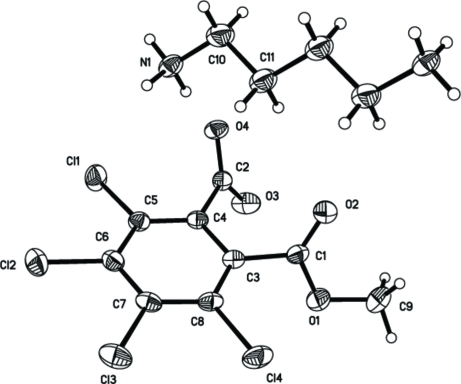
The asymmetric unit of (I), drawn with 30% probability ellipsoids. The symmetry complete cation is shown with unlabeled atoms related by the symmetry operator (-x+1, -y+2, -z+1).

**Fig. 2.**
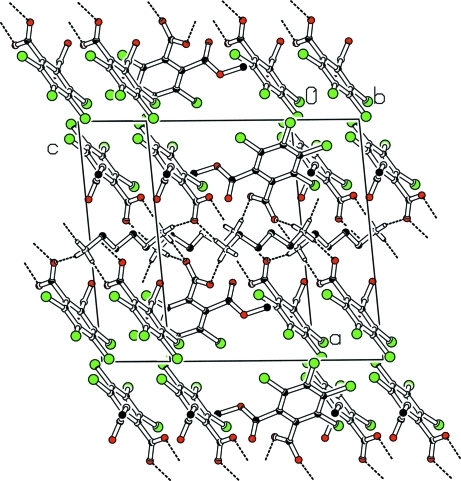
The crystal packing of (I) with hydrogen bonds shown as dashed lines. Only H atoms involved in hydrogen bonds are shown.

## supplementary crystallographic information

### Comment

In the present work, the reaction of 3,4,5,6-tetrachloro-2-(methoxycarbonyl)benzoic acid and butane-1,4-diamine in methanol was expected to yield 4,5,6,7-tetrachloro-2-[4-(4,5,6,7- tetrachloro-1,3-dioxoisoindolin-2-yl)butanyl]isoindoline-1,3-dione. However, the product was the title compound and this may have occurred because of the reduced time and temperature of the reaction. The asymmetric unit of the title compound (I) contains one half of a butane-1,4-diaminium cation and one 3,4,5,6-tetrachloro-2-(methoxycarbonyl)benzoate anions (Fig. 1). In the anion, the mean planes of the methoxycarbonyl and carboxyl groups are aligned at dihedral angles of 64.9 (3) and 58.5 (3) °, respectively with the benzene ring. The bond lengths and angles are in agreement with those which are related in hexane-1,6-diaminium bis[3,4,5,6-tetrachloro-2-(methoxycarbonyl)benzoate](Li, 2011). In the crystal, intermolecular N—H···O hydrogen bonds connect the components into two-dimensional sheets parallel to (100) (Fig. 2 and Table 1).

### Experimental

A mixture of 4,5,6,7-tetrachloroisobenzofuran-1,3-dione (2.86 g, 0.01 mol) and methanol (15 ml) was refluxed for 0.5 h. Then butane-1,4-diamine (0.44 g, 0.005 mol) was added to the above solution and mixed for 20 min at room temperature. The solution was kept at room temperature for 5 d. Natural evaporation gave colourless single crystals of the title compound, suitable for X-ray analysis.

### Refinement

H atoms were initially located from difference maps and then refined in a riding-model approximation with C—H = 0.96–0.97 Å and N—H = 0.89 Å *U*_iso_(H) = 1.2*U*_eq_(C) or 1.5*U*_eq_(N, methyl C).

### Crystal data

**Table:**

C_4_H_14_N_2_^2+^·2C_9_H_3_Cl_4_O_4_^−^	*F*(000) = 732
*M**_r_* = 724.00	*D*_x_ = 1.622 Mg m^−^^3^
Monoclinic, *P*2_1_/*c*	Mo *K*α radiation, λ = 0.71073 Å
Hall symbol: -P 2ybc	Cell parameters from 2612 reflections
*a* = 14.4243 (13) Å	θ = 2.4–26.5°
*b* = 6.1041 (6) Å	µ = 0.81 mm^−^^1^
*c* = 16.9653 (15) Å	*T* = 298 K
β = 97.056 (1)°	Block, colorless
*V* = 1482.4 (2) Å^3^	0.46 × 0.43 × 0.40 mm
*Z* = 2	

### Data collection

**Table:**

Bruker SMART CCD diffractometer	2603 independent reflections
Radiation source: fine-focus sealed tube	1908 reflections with *I* > 2σ(*I*)
graphite	*R*_int_ = 0.024
φ and ω scans	θ_max_ = 25.0°, θ_min_ = 2.4°
Absorption correction: multi-scan (*SADABS*; Bruker, 1997)	*h* = −9→17
*T*_min_ = 0.708, *T*_max_ = 0.738	*k* = −7→7
7307 measured reflections	*l* = −18→20

### Refinement

**Table:**

Refinement on *F*^2^	Primary atom site location: structure-invariant direct methods
Least-squares matrix: full	Secondary atom site location: difference Fourier map
*R*[*F*^2^ > 2σ(*F*^2^)] = 0.036	Hydrogen site location: inferred from neighbouring sites
*wR*(*F*^2^) = 0.096	H-atom parameters constrained
*S* = 1.05	*w* = 1/[σ^2^(*F*_o_^2^) + (0.0373*P*)^2^ + 1.0059*P*] where *P* = (*F*_o_^2^ + 2*F*_c_^2^)/3
2603 reflections	(Δ/σ)_max_ < 0.001
183 parameters	Δρ_max_ = 0.23 e Å^−^^3^
0 restraints	Δρ_min_ = −0.20 e Å^−^^3^

### Special details

**Table:**

Geometry. All e.s.d.'s (except the e.s.d. in the dihedral angle between two l.s. planes) are estimated using the full covariance matrix. The cell e.s.d.'s are taken into account individually in the estimation of e.s.d.'s in distances, angles and torsion angles; correlations between e.s.d.'s in cell parameters are only used when they are defined by crystal symmetry. An approximate (isotropic) treatment of cell e.s.d.'s is used for estimating e.s.d.'s involving l.s. planes.
Refinement. Refinement of *F*^2^ against ALL reflections. The weighted *R*-factor *wR* and goodness of fit *S* are based on *F*^2^, conventional *R*-factors *R* are based on *F*, with *F* set to zero for negative *F*^2^. The threshold expression of *F*^2^ > σ(*F*^2^) is used only for calculating *R*-factors(gt) *etc*. and is not relevant to the choice of reflections for refinement. *R*-factors based on *F*^2^ are statistically about twice as large as those based on *F*, and *R*- factors based on ALL data will be even larger.

### Fractional atomic coordinates and isotropic or equivalent isotropic displacement parameters (Å^2^)

**Table:**

	*x*	*y*	*z*	*U*_iso_*/*U*_eq_	
Cl1	0.27338 (6)	0.79357 (15)	0.17344 (4)	0.0617 (3)	
Cl2	0.10875 (6)	1.10088 (15)	0.19656 (5)	0.0705 (3)	
Cl3	0.01193 (5)	1.07644 (14)	0.34929 (5)	0.0642 (3)	
Cl4	0.07322 (6)	0.72291 (15)	0.47687 (5)	0.0630 (3)	
N1	0.46526 (16)	0.9951 (4)	0.31490 (13)	0.0465 (6)	
H1A	0.4416	0.8603	0.3128	0.070*	
H1B	0.5002	1.0132	0.2756	0.070*	
H1C	0.4189	1.0923	0.3099	0.070*	
O1	0.19109 (14)	0.3094 (3)	0.46777 (12)	0.0559 (5)	
O2	0.32465 (15)	0.4957 (4)	0.48905 (13)	0.0675 (6)	
O3	0.31815 (14)	0.2789 (3)	0.31198 (14)	0.0626 (6)	
O4	0.41355 (12)	0.5599 (3)	0.30166 (11)	0.0459 (5)	
C1	0.24890 (19)	0.4667 (5)	0.45324 (15)	0.0415 (6)	
C2	0.33628 (19)	0.4761 (4)	0.30956 (14)	0.0379 (6)	
C3	0.20896 (17)	0.6149 (4)	0.38674 (15)	0.0376 (6)	
C4	0.25510 (17)	0.6303 (4)	0.31904 (15)	0.0358 (6)	
C5	0.22239 (18)	0.7791 (4)	0.26073 (15)	0.0390 (6)	
C6	0.14757 (18)	0.9170 (4)	0.26928 (16)	0.0428 (7)	
C7	0.10285 (18)	0.9025 (4)	0.33718 (17)	0.0430 (7)	
C8	0.13250 (18)	0.7475 (5)	0.39497 (16)	0.0415 (7)	
C9	0.2212 (2)	0.1710 (6)	0.53489 (19)	0.0662 (9)	
H9A	0.2630	0.0610	0.5195	0.099*	
H9B	0.1679	0.1018	0.5529	0.099*	
H9C	0.2529	0.2581	0.5770	0.099*	
C10	0.5233 (2)	1.0276 (6)	0.39168 (18)	0.0619 (9)	
H10A	0.5461	1.1772	0.3950	0.074*	
H10B	0.5770	0.9309	0.3947	0.074*	
C11	0.4702 (2)	0.9828 (6)	0.46041 (17)	0.0578 (8)	
H11A	0.4477	0.8330	0.4572	0.069*	
H11B	0.4163	1.0791	0.4572	0.069*	

### Atomic displacement parameters (Å^2^)

**Table:**

	*U*^11^	*U*^22^	*U*^33^	*U*^12^	*U*^13^	*U*^23^
Cl1	0.0654 (5)	0.0794 (6)	0.0435 (4)	0.0200 (4)	0.0191 (4)	0.0101 (4)
Cl2	0.0592 (5)	0.0752 (6)	0.0774 (6)	0.0234 (5)	0.0093 (4)	0.0269 (5)
Cl3	0.0443 (4)	0.0644 (5)	0.0856 (6)	0.0176 (4)	0.0143 (4)	−0.0125 (4)
Cl4	0.0541 (5)	0.0812 (6)	0.0597 (5)	0.0003 (4)	0.0309 (4)	−0.0073 (4)
N1	0.0478 (14)	0.0430 (13)	0.0528 (14)	0.0035 (11)	0.0233 (11)	0.0023 (11)
O1	0.0553 (13)	0.0521 (12)	0.0602 (13)	−0.0123 (11)	0.0065 (10)	0.0088 (10)
O2	0.0502 (13)	0.0832 (16)	0.0665 (14)	−0.0169 (12)	−0.0032 (11)	0.0221 (12)
O3	0.0513 (13)	0.0355 (12)	0.1034 (18)	0.0027 (10)	0.0198 (12)	−0.0069 (11)
O4	0.0376 (11)	0.0456 (11)	0.0581 (12)	0.0035 (9)	0.0206 (9)	−0.0009 (9)
C1	0.0385 (16)	0.0471 (17)	0.0407 (15)	−0.0064 (13)	0.0117 (13)	−0.0019 (12)
C2	0.0390 (16)	0.0385 (16)	0.0372 (14)	0.0043 (13)	0.0087 (12)	−0.0014 (11)
C3	0.0320 (14)	0.0382 (15)	0.0433 (14)	−0.0038 (12)	0.0076 (11)	−0.0036 (12)
C4	0.0319 (14)	0.0339 (14)	0.0424 (14)	−0.0005 (11)	0.0077 (11)	−0.0051 (11)
C5	0.0352 (15)	0.0435 (15)	0.0393 (14)	0.0011 (12)	0.0083 (11)	−0.0043 (12)
C6	0.0357 (15)	0.0415 (16)	0.0502 (16)	0.0029 (13)	0.0014 (12)	0.0003 (13)
C7	0.0308 (14)	0.0415 (16)	0.0572 (17)	0.0040 (12)	0.0074 (13)	−0.0096 (13)
C8	0.0335 (14)	0.0487 (17)	0.0448 (15)	−0.0025 (13)	0.0144 (12)	−0.0093 (13)
C9	0.077 (2)	0.062 (2)	0.063 (2)	−0.0074 (19)	0.0209 (18)	0.0136 (17)
C10	0.0485 (19)	0.082 (2)	0.057 (2)	−0.0095 (17)	0.0134 (15)	0.0004 (17)
C11	0.0532 (19)	0.067 (2)	0.0557 (18)	−0.0077 (17)	0.0179 (15)	0.0026 (16)

### Geometric parameters (Å, °)

**Table:**

Cl1—C5	1.735 (3)	C3—C8	1.389 (4)
Cl2—C6	1.711 (3)	C3—C4	1.400 (3)
Cl3—C7	1.719 (3)	C4—C5	1.382 (4)
Cl4—C8	1.725 (3)	C5—C6	1.390 (4)
N1—C10	1.473 (4)	C6—C7	1.390 (4)
N1—H1A	0.8900	C7—C8	1.391 (4)
N1—H1B	0.8900	C9—H9A	0.9600
N1—H1C	0.8900	C9—H9B	0.9600
O1—C1	1.314 (3)	C9—H9C	0.9600
O1—C9	1.441 (4)	C10—C11	1.497 (4)
O2—C1	1.196 (3)	C10—H10A	0.9700
O3—C2	1.234 (3)	C10—H10B	0.9700
O4—C2	1.248 (3)	C11—C11^i^	1.518 (6)
C1—C3	1.504 (4)	C11—H11A	0.9700
C2—C4	1.526 (3)	C11—H11B	0.9700
			
C10—N1—H1A	109.5	C6—C7—C8	119.6 (2)
C10—N1—H1B	109.5	C6—C7—Cl3	119.9 (2)
H1A—N1—H1B	109.5	C8—C7—Cl3	120.6 (2)
C10—N1—H1C	109.5	C3—C8—C7	120.3 (2)
H1A—N1—H1C	109.5	C3—C8—Cl4	120.6 (2)
H1B—N1—H1C	109.5	C7—C8—Cl4	119.1 (2)
C1—O1—C9	116.1 (2)	O1—C9—H9A	109.5
O2—C1—O1	125.0 (3)	O1—C9—H9B	109.5
O2—C1—C3	122.2 (3)	H9A—C9—H9B	109.5
O1—C1—C3	112.7 (2)	O1—C9—H9C	109.5
O3—C2—O4	126.8 (3)	H9A—C9—H9C	109.5
O3—C2—C4	115.5 (2)	H9B—C9—H9C	109.5
O4—C2—C4	117.7 (2)	N1—C10—C11	112.0 (2)
C8—C3—C4	120.5 (2)	N1—C10—H10A	109.2
C8—C3—C1	120.7 (2)	C11—C10—H10A	109.2
C4—C3—C1	118.6 (2)	N1—C10—H10B	109.2
C5—C4—C3	118.5 (2)	C11—C10—H10B	109.2
C5—C4—C2	121.9 (2)	H10A—C10—H10B	107.9
C3—C4—C2	119.6 (2)	C10—C11—C11^i^	112.1 (3)
C4—C5—C6	121.6 (2)	C10—C11—H11A	109.2
C4—C5—Cl1	120.1 (2)	C11^i^—C11—H11A	109.2
C6—C5—Cl1	118.3 (2)	C10—C11—H11B	109.2
C5—C6—C7	119.5 (2)	C11^i^—C11—H11B	109.2
C5—C6—Cl2	120.8 (2)	H11A—C11—H11B	107.9
C7—C6—Cl2	119.7 (2)		
			
C9—O1—C1—O2	3.3 (4)	C4—C5—C6—C7	−1.5 (4)
C9—O1—C1—C3	−175.2 (2)	Cl1—C5—C6—C7	177.3 (2)
O2—C1—C3—C8	−112.4 (3)	C4—C5—C6—Cl2	179.6 (2)
O1—C1—C3—C8	66.2 (3)	Cl1—C5—C6—Cl2	−1.6 (3)
O2—C1—C3—C4	62.3 (4)	C5—C6—C7—C8	−1.2 (4)
O1—C1—C3—C4	−119.2 (3)	Cl2—C6—C7—C8	177.8 (2)
C8—C3—C4—C5	−0.4 (4)	C5—C6—C7—Cl3	178.6 (2)
C1—C3—C4—C5	−175.1 (2)	Cl2—C6—C7—Cl3	−2.5 (3)
C8—C3—C4—C2	−177.3 (2)	C4—C3—C8—C7	−2.2 (4)
C1—C3—C4—C2	8.0 (4)	C1—C3—C8—C7	172.4 (2)
O3—C2—C4—C5	−119.9 (3)	C4—C3—C8—Cl4	178.0 (2)
O4—C2—C4—C5	61.3 (3)	C1—C3—C8—Cl4	−7.5 (3)
O3—C2—C4—C3	56.9 (3)	C6—C7—C8—C3	3.0 (4)
O4—C2—C4—C3	−121.9 (3)	Cl3—C7—C8—C3	−176.8 (2)
C3—C4—C5—C6	2.3 (4)	C6—C7—C8—Cl4	−177.2 (2)
C2—C4—C5—C6	179.1 (2)	Cl3—C7—C8—Cl4	3.1 (3)
C3—C4—C5—Cl1	−176.52 (19)	N1—C10—C11—C11^i^	−179.8 (3)
C2—C4—C5—Cl1	0.3 (4)		

Symmetry codes: (i) −*x*+1, −*y*+2, −*z*+1.

### Hydrogen-bond geometry (Å, °)

**Table:**

*D*—H···*A*	*D*—H	H···*A*	*D*···*A*	*D*—H···*A*
N1—H1C···O3^ii^	0.89	1.85	2.735 (3)	172
N1—H1B···O4^iii^	0.89	1.94	2.823 (3)	174
N1—H1A···O4	0.89	1.88	2.761 (3)	169

Symmetry codes: (ii) *x*, *y*+1, *z*; (iii) −*x*+1, *y*+1/2, −*z*+1/2.
